# Bottom layer absorption coefficients extraction from two-layer phantoms based on crossover point in diffuse reflectance

**DOI:** 10.1117/1.JBO.26.11.117001

**Published:** 2021-11-30

**Authors:** Pavitra S. Rudraiah, Hamootal Duadi, Dror Fixler

**Affiliations:** Bar Ilan University, Faculty of Engineering and Institute of Nanotechnology and Advanced Materials, Ramat Gan, Israel

**Keywords:** diffuse reflectance, penetration depth, optical properties, solid phantom, two-layer, absorption coefficient, top layer thickness, crossover point

## Abstract

**Significance:** Numerous optical imaging and spectroscopy techniques are used to study the tissue-optical properties; the majority of them are limited in information regarding the penetration depth. A simple, safe, easily applicable diagnostic technique is required to get deeper tissue information in a multilayer structure.

**Aim:** A fiber-based diffuse reflectance (DR) technique is used to extract and quantify the bottom layer absorption coefficients in two-layer (2L) tissue-mimicking solid phantoms. We determine the Indian black ink concentrations in a deep-hidden layer that is sandwiched between agar and silicone-based phantom layers.

**Approach:** A fiber-based DR experiment was performed to study the optical properties of the tissue at higher penetration depth, with different fiber core diameters and a constant numerical aperture (0.5 NA). The optimal core diameter of the fiber was chosen by measuring solid phantoms. In 2L phantoms, the thickness of the top layer was kept 5.5 mm with a constant absorption and reduced scattering coefficients ($\mu_{a}=0.045\;{\mathrm{mm}}^{-1}$ and ${\mu_{s}}^{\prime }=2.622\;{\mathrm{mm}}^{-1}$), whereas the absorption coefficients of the bottom layers were varied from 0.014 to $0.037\;{\mathrm{mm}}^{-1}$ keeping the ${\mu_{s}}^{\prime }$ the same as the top layer. A unique crossover point (Cp) was found in the DR intensity profile against distance. We examined the slope before and after the Cp. These two slopes indicate the difference between the optical properties of the top and bottom layers. Our technique got further verification, as we successfully determined the Cp with different Indian black ink concentrations, placed at the junction between the agar and silicone-based phantom layers.

**Results:** The DR measurements were applied to 2L phantoms. Two different slopes were found in 2L phantoms compared to the one-layer (optical properties equal to the top layer of 2L). We extracted the slopes before and after the Cp in the 2L phantoms. The calculated absorption coefficients before the Cp were 0.014±0.0004, 0.022±0.0003, 0.028±0.0003, and $0.036\pm 0.0014\;{\mathrm{mm}}^{-1}$, and the absorption coefficients after the Cp were 0.019±0.0013, 0.013±0.0004, 0.014±0.0006, and $0.031\pm 0.0001\;{\mathrm{mm}}^{-1}$, respectively. The calculated absorption coefficients before the Cp were in good agreement with the optical properties of the bottom layer. The calculated absorption coefficients after the Cp were not the same as the top layer. Our DR system successfully determines the crossover points 12.14±0.11 and 11.73±0.15  mm for 70% and 100% ink concentrations placed at the junction of the agar and silicone layers.

**Conclusions:** In a 2L tissue structure, the Cp depends on the absorption coefficients of top and bottom layers and the thickness of the top layer. With the help of the Cp and the absorption coefficients, one can determine the thickness of the top layer or vice versa. The slope value before the Cp in the DR profile allowed us to determine the absorption properties of the bottom layer instead of having the average behavior of the 2L phantom in the far detection range (11.0 to 17.0 mm).

## 1 Introduction

During the last decades, several models were developed to analyze the reflected light intensity from biological samples. Most of them study the single-layer tissue by describing a single set of optical properties [reduced scattering coefficient (${\mu_{s}}^{\prime }$) and absorption coefficient ($\mu_{a}$)].^1^ Some models study the multilayer tissue structures using different optical parameters depending on the application and which tissue model is of interest.^2^^,^^3^ Many of them are interested in the detailed investigation of the interaction of light in multilayer tissue and the path at which the light is traveling in a complex medium.^4^^–^^6^ Numerous theoretical studies were done to understand the propagation of light in a two-layer (2L) tissue model. A few of the studies support the experimental evidence for the interaction of a photon with a 2L tissue medium using optical tissue-mimicking phantoms.^7^^–^^9^

Ankri et al. showed the experimental confirmation of reflected light intensity profile in 2L solid phantoms using the breaking point between the 2L phantom compared to one-layer (1L) phantom (optical properties equal to the top layer of the 2L phantom). The condition was that when the absorptivity of the top layer was higher than the bottom layer for thin top layer thickness.^10^ In this research, there was no quantitative information about the extraction of optical properties and depth of the incoming photon in the 2L phantom. Alwin et al. showed Monte Carlo simulations for a 2L tissue model with larger top layer thicknesses.^5^^,^^11^ But none of these quantify the optical properties extraction and depth information.

Nossal et al. presented a 2L tissue model for two cases.^12^ In the first case, the absorption coefficient of the bottom layer was greater than the top-layer, and in the second case, the top layer absorption coefficient was much greater than the bottom layer. In both cases, DR intensities (R(r)) were collected as a function of distance (r). In the first case, the reflectance intensity profile showed the average behavior of the 2L tissue model, whereas in the second case, it anticipated the DR profile. It exhibited the two different slope values in the remittance intensity profile in the top and the bottom layers, respectively. The distance at which the reflectance profile showed the intersection between the 2L tissue structure compared to 1L structure was called the crossover point (Cp).^12^^–^^14^ The Cp in the curve confirms the presence of 2L with different absorption coefficients. The Cp is defined in Eq. (1), which is directly proportional to the absorption coefficient of the top ($\mu_{a1}$) layer, bottom ($\mu_{a2}$) layer, and the thickness of the top layer (T):^13^

$$C_{p}\approx T(1+\sqrt{\frac{\mu_{a1}}{\mu_{a2}}}).$$ (1)

Nossal et al. showed simulations for the 2L tissue model in a condition where the bottom layer absorption coefficient was lower than the top layer absorption coefficient ($\mu_{a2}<\mu_{a1}$). They demonstrated that the photons remitted sufficiently far from the illumination point and most likely they moved primarily within the bottom layer.^12^^,^^14^ This condition revealed the deep tissue information from DR intensity profiles, which was collected by scanning the detector fiber on the sample surface over a range of distances. The collected intensity profile plotted as the logarithm of the product of reflected intensity and the square distance [ln ($\text{intensity}\times {\text{distance}}^{2}$)] against distance. The slope was extracted from the linear region of the intensity profile and described in detail in our previous work.^15^ The reduced scattering coefficient (${\mu_{s}}^{\prime }$) is calculated from Eq. (2) using the slope values and the known absorption coefficient ($\mu_{a}$). Equation (2) is approximated under the condition at which the reduced scattering coefficient is much greater than the absorption coefficient ($\mu_{s}^{\prime }\gg \mu_{a}$). The effective attenuation coefficient ($\mu_{\mathrm{eff}}$) is shown in Eq. (2) from Ref. 16:

$$\mu_{\mathrm{eff}}=\sqrt{3\mu_{s}^{\prime }\mu_{a}}.$$ (2)

In this paper, we are using a larger top-layer thickness (5.5 mm) to study the deeper tissue information under the condition when the top layer absorptivity is greater than the bottom layer absorptivity. In far source–detector distances (SDD: 11.0 to 17.0 mm) the majority of the photon migration occurs within the bottom layer. The reemitted photons were from the deeper tissue region.

In the medical diagnostic field, the desire is that a system should provide deep tissue information noninvasively *in vivo* to study abnormalities in the biological system. The more realistic case is the layered tissue characterization that corresponds to the skin, esophagus, intestine, stomach, brain, bladder, etc.^17^^–^^19^

The DR sensing technique is a noninvasive method that can be directly applied to *in vivo* or *in vitro* studies that provide scattering or absorption information at the molecular level.^20^^–^^23^ In this study, we standardize the DR optical setup using different fiber core diameters with a constant numerical aperture (NA). From these, we choose one optimal fiber diameter to determine the Cp between the 2L phantoms compared with the corresponding 1L phantom. We are interested in the extraction and quantification of the hidden layers using the Cp in the tissue-like solid phantoms. The purpose of using solid phantoms is they have stable optical properties and longer shelf life.^24^

We made four 2L samples where we kept the $\mu_{a}$ and ${\mu_{s}}^{\prime }$ constant in the top layer and constant ${\mu_{s}}^{\prime }$ in the bottom layer. But we varied the $\mu_{a}$ of the bottom layer from low to high in these samples. In the case of typical biological tissue, top-layer absorption coefficient is greater than the bottom layer absorption coefficient. The slope from the first region of the DR profile allowed us to determine the absorption properties of the bottom layer instead of having the average behavior of the 2L in the far detection range. Also, a distinct Cp was found in the DR intensity profile for different ink concentrations placed between deep tissue layers.

## 2 Materials and Methods

### 2.1 Experimental Setup

**Fig. 1:**
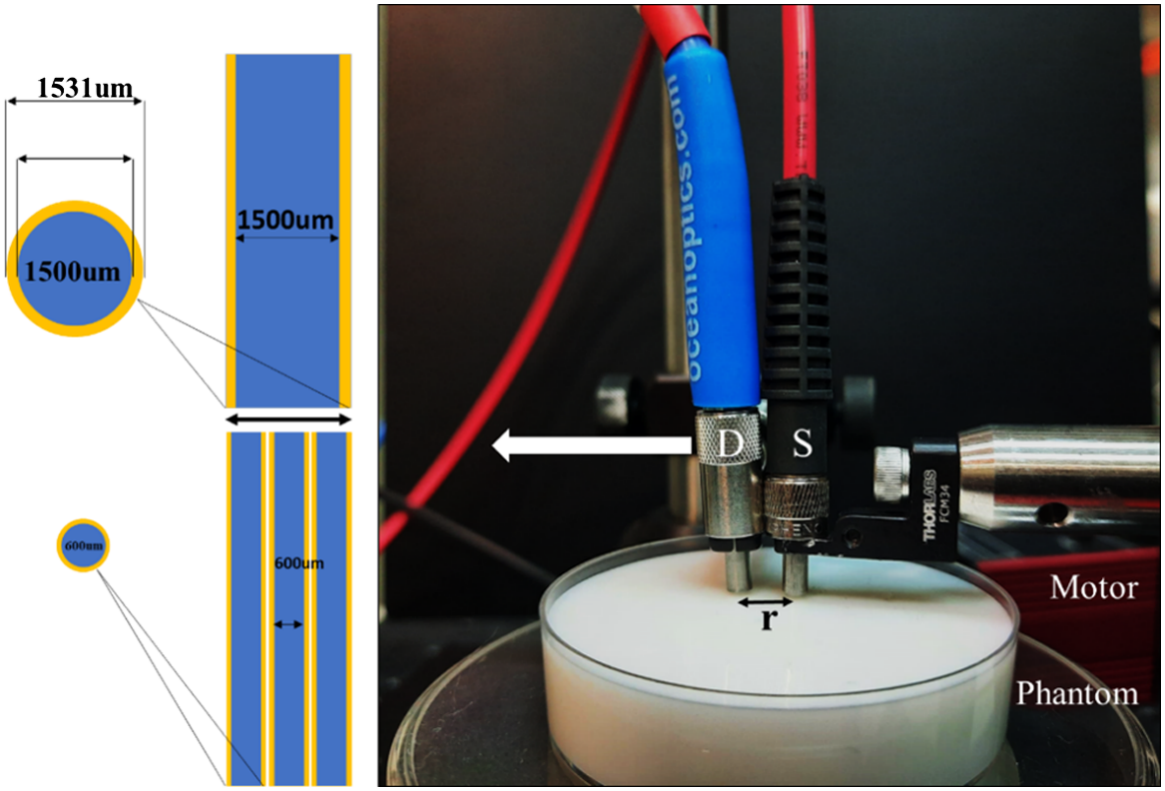
The DR setup using fibers with different core diameters. The source fiber (S) had a 1500 μm core diameter, and the detection fiber (D) core was 400, 600, or 1500  μm. The motor moves the detector fiber (white arrow indicates the direction of movement) with a precise step size according to the fiber cladding. The increase in core diameter will reduce the number of sampling points, illustrated on the left panel. Phantom mimicking the optical properties of tissue, were measured by the different collection fibers.

The DR experimental setup consists of two multimode fibers. One fiber was used as a source fiber with a diameter of 1500  μm and NA of 0.5 (M107L02, Thorlabs, denoted by S in Fig. 1). Another fiber with a 0.5 NA was used to collect the re-emitted signal from the sample (fiber denoted by D in Fig. 1). The light source was a tungsten-halogen lamp (HL-2000-HP-FHSA, Ocean Insight, with 20 W output power). A spectrometer collects the optical measurement (FLAME-T-VIS-NIR-Spectrometer, Ocean Insight). The detector fiber was moved with a precise step size using a stepper motor controller (KST101, Thorlabs).

Before choosing a specific fiber core diameter as a detector fiber, we optimize the DR experimental setup using different fiber core diameters. We used 400, 600, 1500  μm (QP400-1-UV-VIS ocean Insight, M53L02, and M107L02 Thorlabs) fiber cores with the same NA (0.5) for DR measurement to extract the optical properties. The intensity was collected in a step size that matches the fiber clad diameter. Hence, one step from the 1500  μm fiber diameter is equivalent to three steps, from the fiber having a diameter of 600  μm (fiber illustrated in the left panel in Fig. 1). Due to ferrule connectors present in the optical fiber (different manufacturers), the initial distance r between illumination fiber and the detection fiber was ∼9 to 11 mm. At first, the light source and dark spectra were calibrated against a DR standard Spectralon (Newport). After the calibration, the reflected light intensity was measured by moving the detector fiber using a motor controller with a step size of 500, 700, or 1600  μm according to the fiber clad diameters (corresponding to a core of 400, 600, and 1500  μm). The spectra were recorded in the wavelength range from 350 to 1000 nm in each step size. The experiment was repeated with four sequential measurements for a different position on the sample.

### 2.2 Tissue Mimicking Optical Phantom Preparation

We prepared three types of tissue-mimicking solid phantoms with different optical properties: 1L phantoms, 2L phantoms, and 2L phantoms with different ink concentrations between the layers.

In the first type, 1L phantoms were prepared by varying Intralipid (IL) concentrations (0.75%, 1%, 1.25%, 1.5%, and 1.75%). Here, IL (Intralipid 20% emulsion, Sigma-Aldrich, Israel) was used as a scattering component, and 1% agarose powder (agarose, low gelling temperature, Sigma-Aldrich, Israel) converted the solution into gel (agarose will allow solidification of the sample). Double distilled water (DDW) was heated to the temperature of ∼65°C while the 2% agarose was slowly added to distilled water. Once the agarose melted completely, IL and additional DDW were added to the solution and mixed for 1 min with a continuous stir and a mixing temperature of ∼40°C to make a homogeneous solution. Note that the 2% agarose was first melted into half of the amount of the final volume, and then it was diluted to 1% following the additional IL and DDW. The homogeneous solution was poured into tissue culture plates (with a diameter of 60 mm and a height of 10 mm) and cooled under vacuum conditions (to avoid air bubbles).^15^

The 2L phantoms had a constant top layer and varying absorption in the bottom layer. The top layer was prepared with a thickness of 5.5 mm and constant absorption concentration and scattering concentration. The bottom layer of the phantom (10 mm thick) had the same scattering concentration as the top layer and varying absorption concentrations. 1L phantoms were prepared, according to the optical properties corresponding to the top and bottom layers of the 2L phantoms, with a thickness of 10 mm. The absorption coefficient according to wavelengths was measured before adding IL and agar using a spectrophotometer. Reduced scattering coefficient according to wavelengths was measured using an integrating sphere.

We prepared 2L phantoms in that the bottom layer was prepared at first with different absorption concentrations (Indian ink: $0.1\times 10^{-5}$, $0.2\times 10^{-5}$, $0.3\times 10^{-5}$, and $0.4\times 10^{-5}$) with a constant scattering concentration (3% Intralipid). Here, Indian black ink (Royal Talens-490 ml) was utilized as an absorbing component and Intralipid (Intralipid 20% Emulsion, Sigma-Aldrich, Israel) as a scattering component. Again 1% agarose powder was used to convert the solution into gel. DDW was heated to the temperature of ∼65°C while the 2% agarose was slowly added to distilled water. Once the agarose melted completely, ink, Intralipid, and additional DDW were added to the solution and mixed for 1 min with a continuous stir and at a mixing temperature of ∼40°C to make a homogeneous solution. The homogeneous solution was poured into crystallizing dishes (with a diameter of 65 mm and a thickness of 20 mm) and cooled under vacuum conditions (to avoid air bubbles). The top layer (thickness denoted as T) solution (3% IL, and Indian ink: $0.5\times 10^{-5}$) and 1% agarose solution were prepared and added on top of the solid bottom layer at room temperature; the thickness of the top layer was maintained as 5.5 mm. Finally, the 2L phantoms were cooled under vacuum conditions to receive solid 2L phantoms.

The same preparation procedure was carried out for single-layer phantoms with different absorption concentrations (Indian ink: $0.1\times 10^{-5}$, $0.2\times 10^{-5}$, $0.3\times 10^{-5}$, and $0.4\times 10^{-5}$) with a constant scattering concentration (3% IL). The solution was poured into tissue culture plates (with a diameter of 60 mm and a height of 10 mm) then cooled under vacuum conditions.

**Fig. 2:**
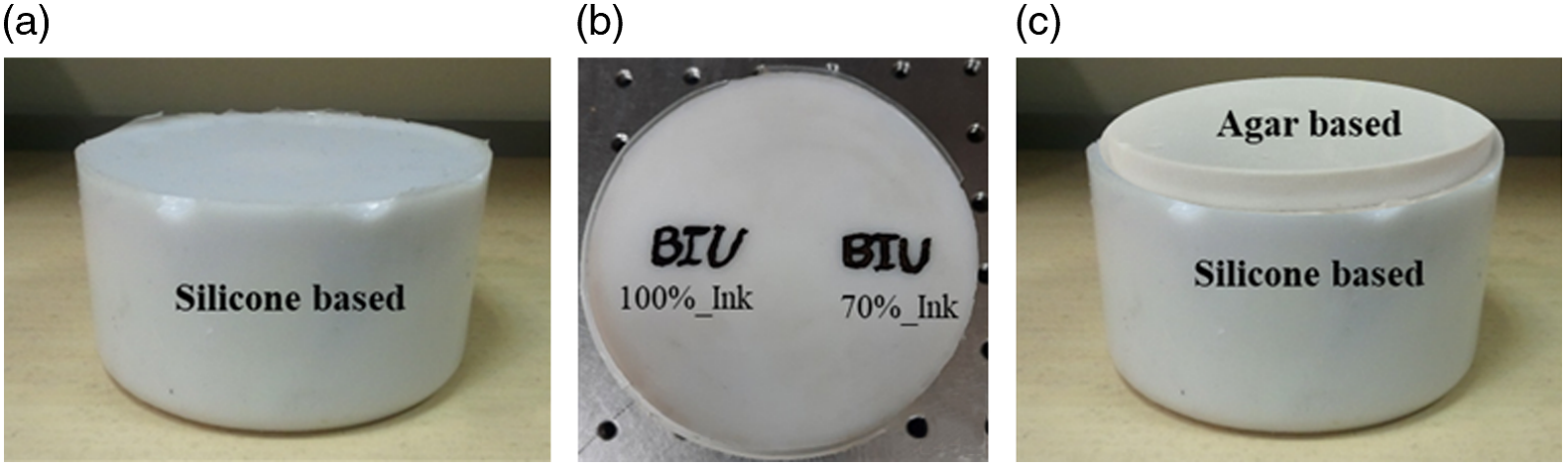
Prepared agar and silicone-based phantoms. (a) Silicone-based phantom, (b) Indian black ink concentrations of 70% and 100% ink introduced by writing the university logo BIU on top of the silicone phantom. (c) 2L phantom made by placing an agar-based phantom on top of silicone phantom, the thickness of the top layer (agar-based phantom) was 5.5 mm.

We prepared synthetic silicone-based phantoms using a P4 silicone rubber base and a p4 silicone activator (Eager Polymers, Chicago, Illinois), along with anatase titanium (IV) oxide and water-soluble nigrosin ink (Sigma-Aldrich, St. Louis, Missouri), for scattering and absorption features, respectively. Components were mixed together to achieve optimal homogeneity: 7 g of titanium (IV) oxide was stirred into the silicone activator by hand. The mixture was placed in a Branson 1200 ultrasonic cleaner (Branson Ultrasonics, Danbury, Connecticut) for 2 h to break apart coagulated titanium (IV) oxide particles. In a separate container, 1 ml of nigrosin solution ($1.5\;g/1\;H_{2}O$) was added to the silicone base and mixed at 2000 to 2500 RPM with a plunge mixer (Freeman Manufacturing & Supply Company, Avon, Ohio) for 5 min. The titanium (IV) oxide suspension was mixed into the nigrosin and silicone base mixture. All components were then mixed for an additional 2 min with the plunge mixer and immediately placed into a Gas Vac II industrial vacuum degassing unit (Freeman Manufacturing & Supply Company, Avon, Ohio). The phantom mixture sat in the degassing chamber for ∼2  min until a pressure of −29  mmHg was achieved and bubbles began to collapse.^25^ Then, the mixture was returned to normal atmospheric pressure, the chamber was vented, and the containers were removed and placed on a flat surface [Fig. 2(a)]).

We wrote the university logo “BIU” from Indian black ink concentrations of 70% and 100% ink on top of the prepared silicone phantom [Fig. 2(b)] using a painting brush. Once the ink was dried completely, the agar-based top layer phantom [Fig. 2(c) top layer: 3% IL and $0.5\times 10^{-5}$ ink, thickness 5.5 mm] was placed carefully on top of the silicone phantom.

## 3 Results and Discussion

### 3.1 Extracted Reduced Scattering Coefficients From 1L Phantoms

A fiber-based DR measurement was performed on five solid phantoms made with different IL concentrations, to extract the reduced scattering coefficients using three fiber core diameters (400, 600, and 1500  μm).

**Fig. 3:**
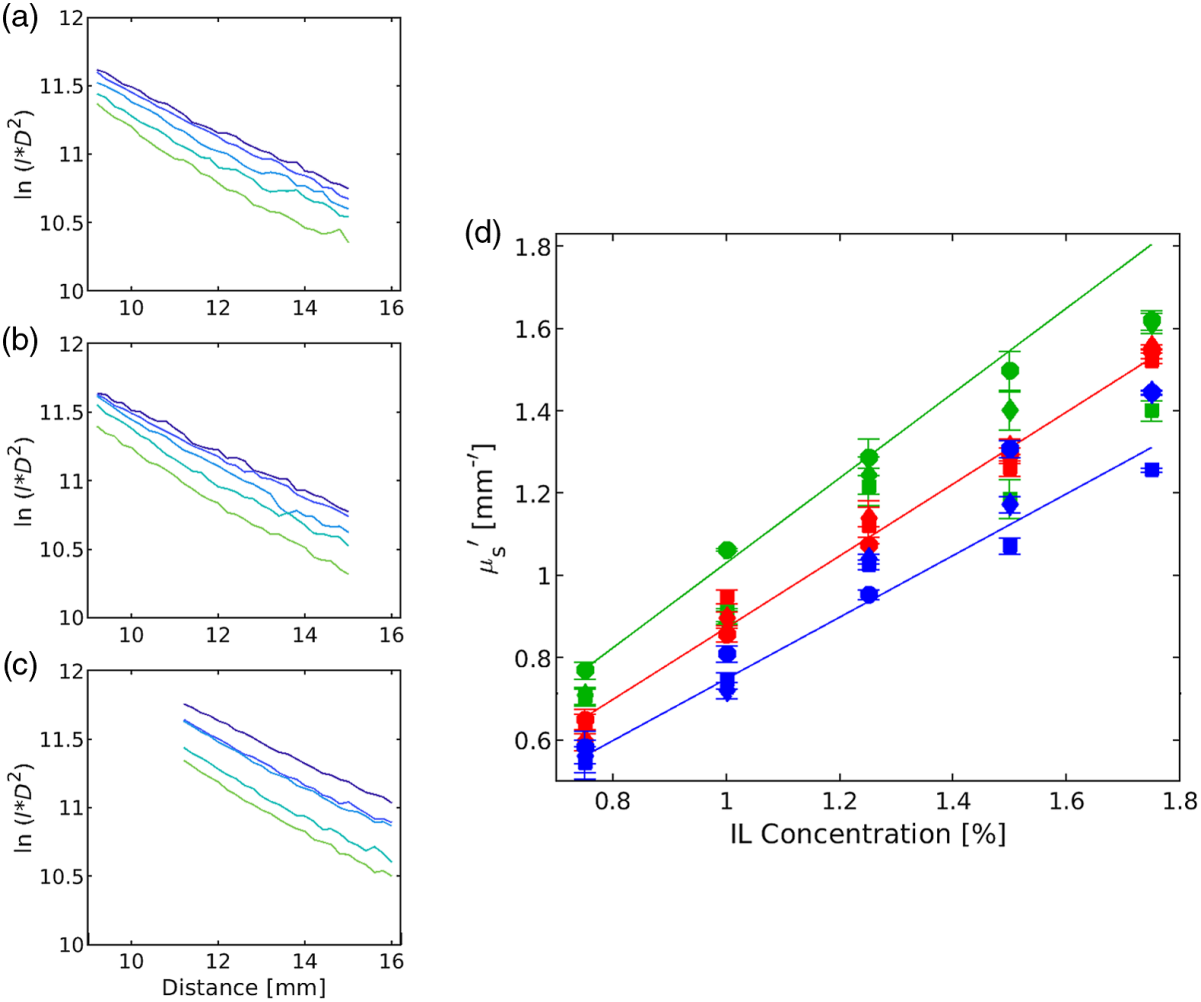
Extracted reduced scattering coefficient as a function of different Intralipid concentrations using different fiber core diameters. Measured diffuse reflection from phantoms made with different IL concentrations (0.75%, 1%, 1.25%, 1.5%, and 1.75% represented by a line with the color dark blue, blue, aqua, green, and light green) as a function of the distance (r) at a wavelength 650 nm using fiber with core of (a) 400, (b) 600 and (c) 1500  μm. (d) Calculated ${\mu_{s}}^{\prime }$ from the slopes, extracted from the curves in (a)–(c) as a function of different IL concentrations with a fiber core diameter of 400, 600, 1500  μm (square, diamond, and filled circle) compared the reduced scattering coefficient to integrating sphere (solid lines) data at different wavelengths: 550, 650, and 750 nm correspond to green, red, and blue. Error bar indicates the standard deviation ($<0.1\;{\mathrm{mm}}^{-1}$).

The collected diffusely reflected intensity from the three fibers shown in Figs. 3(a)–3(c) is the logarithmic of the product between the measured intensity and square distance $\left[\ln(I\times D^{2})\right]$ against distance, at 650 nm, for five IL concentrations (0.75%, 1%, 1.25%, 1.5%, and 1.75%). Figures 3(a)–3(c) correspond to the collected DR intensity from the fiber core diameter 400, 600, and 1500  μm, respectively. DR experiments were done to calibrate the intensity collected by different fiber core diameters with a constant NA.

The slope was extracted from the linear region of the intensity profile from each phantom, where the slope increases with the IL concentrations. From the obtained slope, we calculated the reduced scattering coefficient for each phantom using Eq. (2) with the knowledge of absorption coefficient (here, IL and water are the absorbing components). Figure 3(d) shows the extracted reduced scattering coefficient from five IL concentrations using three fiber core diameters 400, 600, 1500  μm (square, diamond, and filled circle) at wavelengths of 550, 650, and 750 nm (green, red, and blue). The extracted ${\mu_{s}}^{\prime }$ values from three fiber core diameters at 650 nm are shown in Table 1.

**Table 1:** Extracted ${\mu_{s}}^{\prime }$ using three fiber core diameters at 650 nm.

Intralipid Concentrations (%)	400 μm ${\mu_{s}}^{\prime }$ (${\mathrm{mm}}^{-1}$)	600 μm ${\mu_{s}}^{\prime }$ (${\mathrm{mm}}^{-1}$)	1500 μm ${\mu_{s}}^{\prime }$ (${\mathrm{mm}}^{-1}$)	Integrating sphere ${\mu_{s}}^{\prime }$ (${\mathrm{mm}}^{-1}$)
0.75	0.641 ± 0.035	0.599 ± 0.012	0.651 ± 0.042	0.656
1.0	0.950 ± 0.002	0.897 ± 0.034	0.857 ± 0.034	0.874
1.25	1.123 ± 0.014	1.139 ± 0.033	1.075 ± 0.016	1.092
1.5	1.261 ± 0.002	1.314 ± 0.008	1.293 ± 0.047	1.311
1.75	1.523 ± 0.005	1.555 ± 0.005	1.547 ± 0.002	1.529

The extracted reduced scattering coefficient values are in good agreement with the integrating sphere data [solid line in Fig. 3(d)]. We calculated the correlation coefficients for the ${\mu_{s}}^{\prime }$ from the different fiber core diameters. The values are 400  μm: 0.990758, 600  μm: 0.996158, and 1500  μm: 0.999251. However, the closest fit between integrating sphere values and extracted values from the DR system is for a fiber core of 600 and 1500  μm. Hence, we used these fibers for the next 2L phantoms study.

### 3.2 Crossover Point Determination in Two-Layer Phantoms

DR measurements were performed on four 2L tissue-mimicking solid phantoms to extract the optical properties from 2L structures. We kept a constant top layer absorption and reduced scattering coefficients. The reduced scattering coefficient of the bottom layer was the same as the top layer but varying the bottom layer absorption coefficients (typical biological tissue case: top-layer absorption coefficient is greater than the bottom layer absorption coefficient). The total thickness of the 2L phantoms was 15.5 mm, where the top-layer thickness was maintained 5.5 mm and the bottom layer was 10 mm for all four phantoms. The sample preparation procedure is detailed in Sec. 2.3.

**Fig. 4:**
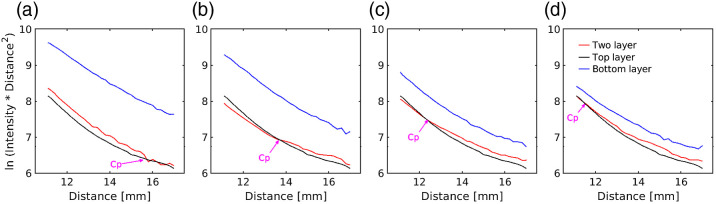
Diffuse reflected intensity profile and crossover point in 2L phantoms (red lines) compared to the 1L phantoms corresponding to the top and bottom layers (black and blue lines) at 650 nm. 2L phantoms are made with constant absorption concentration ($0.5\times 10^{-5}$) and scattering concentration (3% IL) in the top layer, and the bottom layers are made with different absorption concentrations (Indian ink: $0.1\times 10^{-5}$, $0.2\times 10^{-5}$, $0.3\times 10^{-5}$, and $0.4\times 10^{-5}$) with a scattering concentration (3% IL) the same as the top layer. The thickness of the top layer was 5.5 mm, and the bottom layer was 10 mm. The optical coefficients of the 2L phantoms and determined crossover points are: (a) ${\mu_{s1}}^{\prime }=2.622\;{\mathrm{mm}}^{-1}$, $\mu_{a1}=0.045\;{\mathrm{mm}}^{-1}$, ${\mu_{s2}}^{\prime }=2.622\;{\mathrm{mm}}^{-1}$, $\mu_{a2}=0.014\;{\mathrm{mm}}^{-1}$, and Cp is 15.8±0.2  mm, (b) ${\mu_{s1}}^{\prime }=2.622\;{\mathrm{mm}}^{-1}$, $\mu_{a1}=0.045\;{\mathrm{mm}}^{-1}$, ${\mu_{s2}}^{\prime }=2.622\;{\mathrm{mm}}^{-1}$, $\mu_{a2}=0.022\;{\mathrm{mm}}^{-1}$, and Cp is 13.6±0.4  mm, (c) ${\mu_{s1}}^{\prime }=2.622\;{\mathrm{mm}}^{-1}$, $\mu_{a1}=0.045\;{\mathrm{mm}}^{-1}$, ${\mu_{s2}}^{\prime }=2.622\;{\mathrm{mm}}^{-1}$, $\mu_{a2}=0.029\;{\mathrm{mm}}^{-1}$, and Cp is 12.4±0.3  mm, and (d) ${\mu_{s1}}^{\prime }=2.622\;{\mathrm{mm}}^{-1}$, $\mu_{a1}=0.045\;{\mathrm{mm}}^{-1}$, ${\mu_{s2}}^{\prime }=2.622\;{\mathrm{mm}}^{-1}$, $\mu_{a2}=0.037\;{\mathrm{mm}}^{-1}$, and Cp is 11.4±0.6  mm.

Figures 4(a)–4(d) show the logarithm of the product of collected diffuse reflectance (DR) intensity and square distance [ln ($\text{intensity}\times {\text{distance}}^{2}$)] versus distance. The 2L (red line) phantoms were compared with the 1L phantoms with the optical properties corresponding to the top layer (black line) and bottom layer (blue line) with a thickness of 10 mm. The optical properties of the top layer are ${\mu_{s1}}^{\prime }=2.622\;{\mathrm{mm}}^{-1}$, $\mu_{a1}=0.045\;{\mathrm{mm}}^{-1}$, and the bottom layer have the same scattering ${\mu_{s2}}^{\prime }=2.622\;{\mathrm{mm}}^{-1}$ and absorption of: $\mu_{a2}=0.014\;{\mathrm{mm}}^{-1}$, $\mu_{a2}=0.022\;{\mathrm{mm}}^{-1}$, $\mu_{a2}=0.029\;{\mathrm{mm}}^{-1}$, and $\mu_{a2}=0.037\;{\mathrm{mm}}^{-1}$ corresponding to Figs. 4(a)–4(d). The distance at which the 2L phantom intersects the top layer phantom is called the crossover point (Cp). In 2L phantoms, the DR intensity profile shows two different slopes before the Cp and after the Cp.

**Fig. 5:**
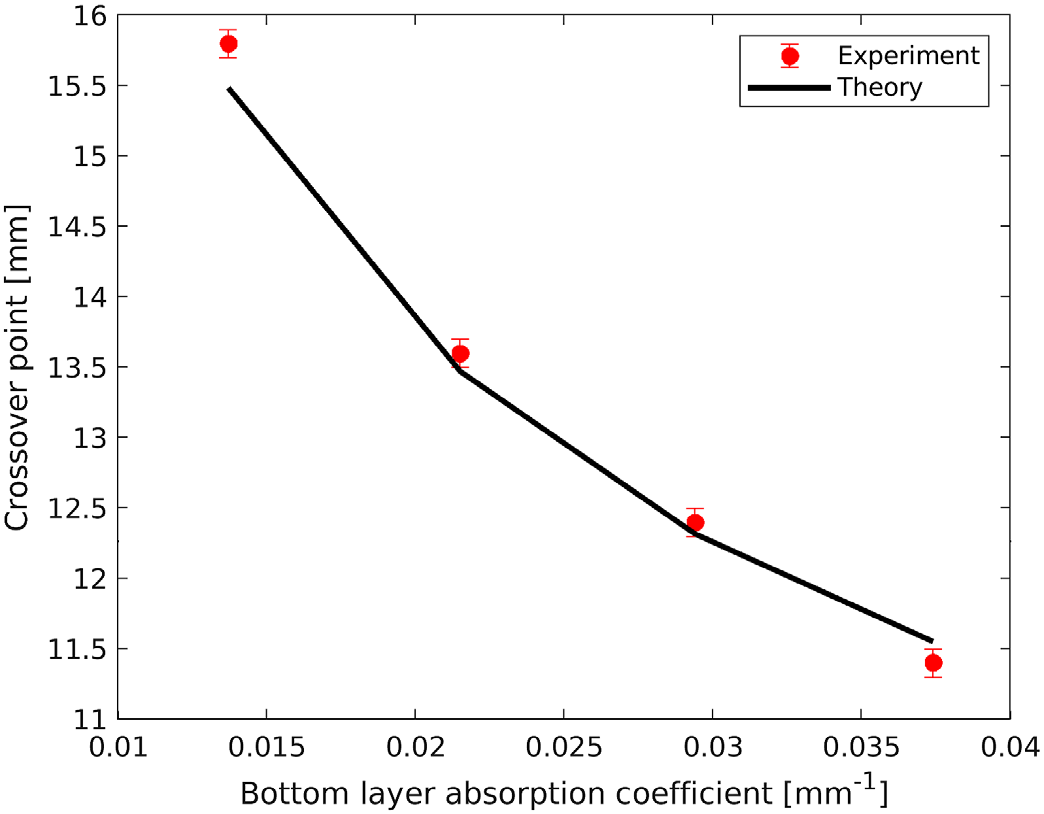
The extracted crossover point from 2L phantoms with varying absorption in the bottom layer at 650 nm. The crossover point was determined using DR measurements (filled red circles) from 2L phantoms compared with the 1L phantoms from Figs. 4(a)–4(d) and the calculated Cp (black line) using Eq. (1). The error bar indicates the standard deviation (<0.0014  mm).

The Cp depends on the absorption coefficients of the top and bottom layers in addition to the thickness of the top layer [according to Eq. (1)]. We use the thickness of the top layer of 5.5 mm and calculate the crossover point from the top and bottom layer’s absorption coefficient (line in Fig. 5). We compare the measured crossover points (red circles in Fig. 5) from Figs. 4(a)–4(d) to the calculated Cp (black line in Fig. 5) according to Eq. (1). The extracted Cps from DR measurements are in good agreement with the calculated values. As the absorption of the top layer increases, the Cp decreases.

Next, we examined the slopes before and after the Cp. In the DR intensity profile, we took a linear fit before the Cp point (first line in Table 2) and after the Cp (second line in Table 2). From these slopes, we calculated the absorption coefficient according to Eq. (2). The third and fourth lines in Table 2 are the extracted $\mu_{a}$ from the slopes in the first and second lines, respectively. The extracted $\mu_{a}$ from the experiments compared to the measured values from the spectrophotometer before the Cp (third and fifth lines in Table 2) are in good agreement. The extracted $\mu_{a}$ after the Cp in Figs. 4(a)–4(d) was not the same as the top layer (fourth and sixth lines in Table 2). For large SDDs (11.0 to 17.0 mm), there is a high chance that the majority of the photon migration occurs within the bottom layer due to longer trajectories, and the re-emitted photons were from the deeper layer. This crossover point can be used as a diagnostic fingerprint of *in vivo* biological systems.

**Table 2:** Calculated $\mu_{a}$ from 2L phantom at 650 nm.

Cp (mm)	Fig. 3(a)	Fig. 3(b)	Fig. 3(c)	Fig. 3(d)
	15.8 ± 0.2	13.6 ± 0.4	12.4 ± 0.3	11.4 ± 0.6
Slope before the Cp	−0.333 ± 0.005	−0.417 ± 0.003	−0.473 ± 0.003	−0.531 ± 0.011
Slope after the Cp	−0.386 ± 0.015	−0.314 ± 0.005	−0.334 ± 0.008	−0.493 ± 0.001
Extracted $\mu_{a}$ (${\mathrm{mm}}^{-1}$) before the Cp	0.014 ± 0.0004	0.022 ± 0.0003	0.028 ± 0.0003	0.036 ± 0.0014
Extracted $\mu_{a}$ (${\mathrm{mm}}^{-1}$) after the Cp	0.019 ± 0.0013	0.013 ± 0.0004	0.014 ± 0.0006	0.031 ± 0.0001
$\mu_{a}$ (${\mathrm{mm}}^{-1}$) of bottom layer from spectrophotometer	0.014	0.022	0.029	0.037
$\mu_{a}$ (${\mathrm{mm}}^{-1}$) of top layer from spectrophotometer	0.045	0.045	0.045	0.045

### 3.3 Determination of Indian Black Ink Concentrations in Two-Layer Phantom

**Fig. 6:**
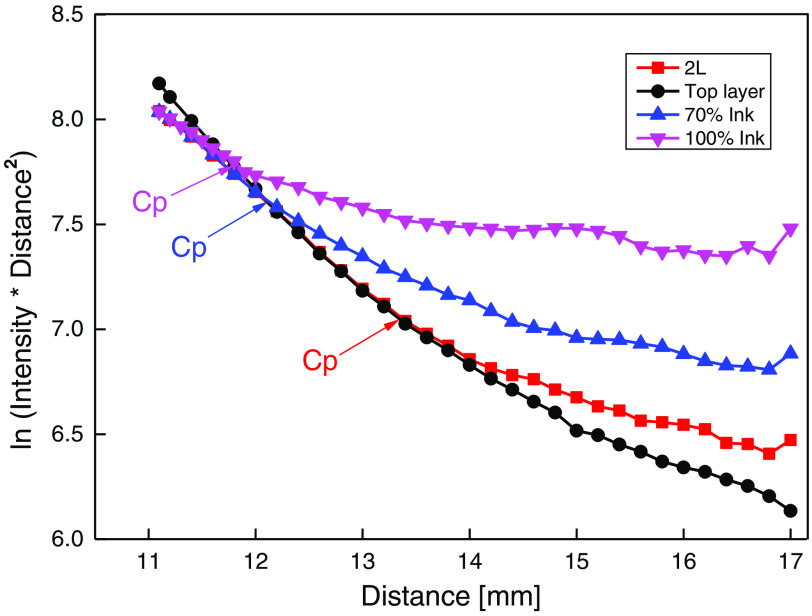
DR intensity profile for 2L (agar and silicone-based) phantom. The obtained crossover points for different ink concentrations at 650 nm. The DR intensity plotted as a logarithm of the product of collected intensity and the square distance versus distance; The top-layer agar-based phantom (black-filled circles). Agar and the silicone-based 2L phantom formed by placing an agar phantom on top of the silicone phantom (red squares). Ink concentrations of 70% ink (blue upward-pointing triangles) and 100% ink (magenta downward-pointing triangles) were introduced (university logo BIU) between the agar and silicone-based 2L phantom.

A DR measurement was performed to determine the ink concentrations in the 2L phantom. The phantom preparation procedure was explained in Sec. 2.3 (Fig. 2). Fiber-based DR experiments were done on the 1L phantoms made with the optical properties equal to the top layer (agar-based) phantom and bottom layer (silicone-based) phantom. The DR intensity was collected from the 2L (agar and silicone-based) phantom before introducing the ink concentrations (red squares in Fig. 6). Then they were collected again after the introduction of ink concentrations at the junction between the 2L phantom (blue upward-pointing triangles for 70% ink and magenta downward-pointing triangles for 100% ink). The plot is the logarithm of the product of collected DR intensity and square distance [ln ($\text{intensity}\times {\text{distance}}^{2}$)] versus distance compared to a 1L (optical properties equal to the top layer agar-based) phantom (black filled circles). We saw different crossover points for 70% ink (blue upward-pointing triangles) and 100% ink (magenta downward-pointing triangles). The bottom layer data is not shown in Fig. 6.

The red Cp between the top layer phantom and the 2L phantom is at a distance of 13.4±0.1  mm. The Cp for the phantom with 70% and 100% ink are at a distance of 12.14±0.11  mm (blue Cp) and 11.73±0.15  mm (magenta Cp), respectively. From Fig. 5, the crossover point and absorption coefficient relation suggest that as the absorption coefficient increases (ink concentration), the crossover point decreases. A decrease in crossover point verifies the presence of higher ink concentrations in the phantom. It confirms the presence of different ink concentrations at the bottom layer. The photons arrived at the bottom layer in the 2L phantom. In typical biological tissue cases such as skin, stomach, bladder brain, esophagus, intestine, etc, this Cp may be used as a diagnostic fingerprint.

## 4 Conclusion

Fiber-based DR experiments were demonstrated to extract the optical properties from 1L tissue-mimicking solid phantoms using different fiber core diameters with a constant NA. We optimized the experimental setup using different fiber core diameters. We chose one best fiber core diameter to collect the diffusely reflected intensity from 2L solid phantoms. The optical properties are calculated from 2L phantoms by extracting the slope values before and after the crossover point in the DR profile. The calculated optical properties before the crossover point are in good agreement with the spectrophotometer data of the bottom layer.

The DR intensity was collected far from the illumination point, hence the majority of photons reached the bottom layer in 2L phantoms. It confirms that our system is able to extract optical properties behind a 5.5 mm thick phantom. In the far detection range, the DR intensity profile allowed us to determine the absorption of the bottom layer instead of the average behavior of the two layers. We successfully determine the pigment concentrations in a 2L phantom with a crossover point model.

The DR measurement is a noninvasive, inexpensive method. It can provide an objective assessment of the pigment concentrations, abnormalities, etc., in a deeper tissue region. The crossover point acts as a diagnostic fingerprint in a multilayer tissue model. In future, we will apply our DR technique to study the optical properties in multilayer tissues.
